# Optimization of Electrolytes with Redox Reagents to Improve the Impedimetric Signal for Use with a Low-Cost Analyzer

**DOI:** 10.3390/bios13120999

**Published:** 2023-11-27

**Authors:** Yu-Hsuan Cheng, Charmi Chande, Zhenglong Li, Niranjan Haridas Menon, Sreerag Kaaliveetil, Sagnik Basuray

**Affiliations:** Otto H. York Department of Chemical and Materials Engineering, New Jersey Institute of Technology, 323 Dr Martin Luther King Jr Blvd, Newark, NJ 07102, USA

**Keywords:** impededance spectroscopy, electrochemical detection, redox probes, low-cost analyzer

## Abstract

The most well-known criterion for POC devices is ASSURED, and affordability, i.e., using low-cost instrumentation, is the most challenging one. This manuscript provides a pathway for transitioning ESSENCE, an impedance-based biosensor platform, from using an expensive benchtop analyzer—KeySight 4294A (~$50k)—to using a significantly portable and cheaper USB oscilloscope—Analog Discovery 2 (~$200) —with similar sensitivity (around 100 times price difference). To achieve this, we carried out a fundamental study of the interplay between an electrolyte like potassium chloride (KCl), and an electrolyte buffer like phosphate buffered saline (PBS) in the presence and absence of a redox buffer like ferro/ferricyanide system and ([Ru(bpy)_3_]^2+^). Redox molecules in the electrolyte caused a significant change in the Nyquist curve of the impedance depending on the redox molecule type. The redox species and the background electrolyte have their own RC semicircles in the Nyquist curve, whose overlap depends on the redox concentration and electrolyte ionic strength. We found that by increasing the electrolyte ionic strength or the redox concentration, the RC semicircle moves to higher frequencies and vice versa. Importantly, the use of the buffer electrolyte, instead of KCl, led to a lower standard deviation and overall signal (lesser sensitivity). However, to achieve the best results from the biorecognition signal, we chose a buffered electrolyte like PBS with high ionic strength and lowered the redox probe concentrations to minimize the standard deviation and reduce any noise from migrating to the low-cost analyzer. Comparing the two analyzers shows similar results, with a lowered detection limit from the low-cost analyzer.

## 1. Introduction

Biosensors have been used as point-of-care (POC) devices due to their potential for biomedical applications across diverse areas, from treatment and diagnosis to prevention [1,2,3,4,5]. Biosensors typically are a two-system process where the first system consists of a biorecognition molecule that allows for specific biochemical reactions for target capture, and then the signal transducer converts this capture signal into a detectable signal using different methods like optical, electrical, or thermal [6]. Most research focused on biorecognition reactions to enhance the sensor’s target-capturing ability, such as developing new antibodies [7,8], molecule implant polymers [9,10,11,12], aptamers of signal strand DNA/RNA [13,14], and proteins [15,16]. These researchers have demonstrated significantly higher sensitivity and selectivity using the modified capture molecules [17,18,19]. For example, Almirola et al. [20] developed a molecule implant polymers as a capturing probe using poly(o-phenylenediamine) on an SPE through electropolymerization. They obtained a LoD of 1 ng/dL and a detection ranging from 1 to 15.7 ng/dL for testosterone levels.

It is important to note that electrochemical-based biosensors that do not require labels have gathered tremendous research attention. This stems from their perceived advantages of multi-target detection, fast detection times, and easy manufacturing processes. Finally, these allow for the development of miniaturized portable POC devices [21]. The high sensitivity and selectivity have also led to significant improvement in the detection limit [22]. Compared to other detecting methods, such as surface plasmon resonance [23] and fluorescence [24], they require less bulky equipment in the field.

In electrochemical methods, electrochemical impedance spectroscopy (EIS) is a powerful tool that allows the analysis of interfacial biorecognition events for a label-free biosensor. For an applied perturbing sinusoidal voltage signal (~mV), the current response is measured with the impedance calculated as the ratio between the voltage and current phasor. The binding of the target molecule to the capture molecule at the electrode surface leads to perturbations in the impedance signals [25,26,27,28]. This perturbation can be quantified by comparing the initial and post-impedance signals. However, these electrochemical biosensors have disadvantages like attenuated signals resulting from self-assembled biomolecule layers and non-specific adsorption onto the electrode surface, which can substantially reduce sensitivity and selectivity [29].

Studies to enhance the label-free electrochemical biosensor’s performance have been widely carried out, and these include using cheap carbon-based transducers [30], nanoparticle usage, redox probes studies, or changing the electrode shape [31]. Of all of the different enhancements, the redox probes are the most commonly used to enhance the label-free biosensor, called a Faradaic sensor. The redox probe adds a small electrochemically sensitive compound to the bulk electrolyte during the measurement. The accessible redox probe reduces or oxidizes at the electrode surface, generating the Faradaic current near/at the location of the biorecognition event. Thus, the change in the impedimetric signal due to the biorecognition event is significantly enhanced in the presence of redox probes. However, the electrolyte properties like the ionic strength, pH, and cation or anion type affect the interactions of the redox molecules with the surface, significantly affecting the impedimetric signal for Faradaic sensors [32]. Lacina et al., showed that the charge of immobilized molecules on the electrode surface significantly affects the measured signals [32,33]. Controlling the bulk concertations of ferro/ferricyanide phosphate buffer with 150 mM NaCl, Lacina et al., showed that specific redox probe concentrations significantly influenced the overall signal response of their electrochemical system. This signal enhancement can be seen in multiple applications. Lin et al. [34] developed a hydrogel patch with a conducting poly(3,4-ethylenedioxythiophene nanocomposite layer to detect the glucose with ferricyanide redox probe enhancement and found it provided not only a higher signal response, but also great electrochemical stability with multiple measurements cycle under 0.1 M potassium chloride (KCl). Rengaraj et al. [35] developed a screen-printed paper sensor to detect the bacteria under the 5 mM redox with fer-ri/ferrocyanide redox couple. Salahandish et al. [36] developed a portable sensor platform for less than $40 and claimed it could detect COVID-19 in 90 s, and showed that the lower redox concentration has a higher sensor response.

In our previous studies [37,38,39], we developed a biosensor platform called ESSENCE. ESSENCE is an electrochemical sensor that uses a shear-enhanced, flow-through manoporous capacitive electrode. In brief, a top and bottom three-dimensional interdigitated micro-electrode array (NP-µIDE) sandwiches a microfluidic channel packed with nano-ordered, tunable-porosity material with grafted target-specific probes. This platform has detected ssDNA [38], protein biomarkers [38,39], and emerging chemical contaminations (PFOS) [40]. Furthermore, we recently showed that using an automatic fluidic control system allows us to take advantage of the analyte flow through the porous layer, leading to enhanced shear forces, mitigating non-specific adsorption, and tremendously increasing selectivity. However, no significant optimizations were carried out regarding the background electrolyte or redox probe that would allow us to enhance the sensitivity and selectivity of ESSENCE.

In this paper, we further improved the sensitivity and selectivity of the ESSENCE platform. We looked at different redox molecules like Tris(bipyridine)ruthenium(II) ([Ru(bpy)_3_]^2+^) and ferro/ferricyanide([Fe(CN)_6_]^4−^ and [Fe(CN)_6_]^3−^) in different buffers like phosphate buffer (PBS) and KCl. Further, we compared impedimetric results under pH control and non-pH control systems with different redox probe pairs and electrolytes to fundamentally understand the impedimetric signal that allowed us to optimize the background electrolyte for maximum sensitivity. The optimized electrolyte/redox probe system allows us to use a cheaper alternative to the much more expensive benchtop 4294A precision impedance analyzer (~100,000 $). Thus, this allows us to use ESSENCE as an affordable POC sensing platform.

## 2. Materials and Methods

### 2.1. Reagents and Instruments

Standard glass slides (1304G) of size 25 mm × 75 mm × 1 mm were from Globe Scientific Inc. (Mahwah, NJ, USA). Tris(bipyridine)ruthenium(II) chloride was from PNNL. PBS and KCL was from VWR (Radnor, PA, USA) The DI water used in the experiments was obtained from a Milli Q Direct 8 Water Purification System (MilliporeSigma, Burlington, MA, USA). Double-sided tapes (ARcare^®^ 90106NB) with polyester film and MA-69 medical-grade acrylic pressure-sensitive adhesives on both sides were obtained from ARcare, Augusta, AR, USA. The thickness of the tape was 140 μm, including the PP layer and the two adhesives. The 4294A Precision Impedance Analyzer was from Keysight Technologies. The Analog Discovery 2 with impedance analyzer was from Digilent, Pullman, WA, USA. The carboxylic acid-functionalized short single-walled carbon nanotubes (SWCNT, 98%+) were acquired from US Research Nanomaterials Inc., Houston, TX, USA. Fabrication of the top and bottom microelectrode glass slide was carried out in the Nano-fabrication facility at CUNY Advanced Science Research Centre, New York, NY, USA. Sequences of the probe-DNA (pDNA) and target-DNA (TDNA)oligo were 5′-/5AmMC6/CGTCCAAGCGGGCTGACTCATCAAG-3′ and 5′-CTT GATGAGTCAGCCCGCTTGGACG-3′, respectively, and they were acquired from Integrated DNA Technologies (IDT). The fully automatic fluidic controlling system was purchased from Labsmith Inc., Livermore, CA, USA. All the PBS used in this chapter was at pH 7.4.

### 2.2. Microfluidic ESSENCE Chip Fabrication and Functional of SWCNT

The microfluidic chip called ESSENCE was fabricated exactly from our previous publications, 86, 159. In brief, the double-sided tape was sandwiched between two standard glass slides with gold microelectrodes. The fabrication details and packing protocol were according to our previous papers [38]. In the next step, a double-sided polyester tape (ARcare^®^ 90106NB) was cut into a rectangular shape and sandwiched between the two glass slides containing the electrodes. Finally, three μL of the functionalized SWCNT packing solutions were pipetted into the channel before closing the device. The amount of the solution used was 1.5 µl, which was added twice into the channel. Subsequently, the solution was left to evaporate, forming a highly packed microfluidic channel between two microelectrodes.

### 2.3. Functional SWCNT Packing

The method of SWCNT functionalization was also similar to our previous method with the optimized procedure [37,38,39], with the exception that Sulfo-NHS was used instead of N-Hydroxysuccinimide. In brief, 50 mg of the SWCNT–COOH suspension was rinsed with 0.1 M MES three times. The standard EDC–NHS two-step coupling reaction was used to activate SWCNT for 20 min. Next, the SWCNT–COOH was vortexed/washed thrice in 1× PBS (pH 7.4) after activation. After washing, the ssDNA oligo probe with 1× PBS solution was immediately added to the activated SWCNT–COOH. The Eppendorf was then rotated and incubated at room temperature overnight. After incubation, the coupling SWCNT was washed in 1× PBS three times to ensure the ssDNA oligo probe or capturing antibody was washed away. Finally, the functionalized SWCNT was mixed with 300 μL 1× (pH 7.4) PBS solution and stored at 4 °C. The functionalized SWCNT was used over months.

### 2.4. Detection Protocol

The detection protocol was according to our previous studies [37,38,39]. In brief, it consisted of three steps, namely, (1) the initial/priming step, (2) the incubation step, and (3) the rinsing step. The chip was first treated with an initial/priming step before the start of the detection protocol. Next, the DNA sample was loaded into the sample chamber on the platform, and the auto-valve system transported it to the ESSENCE chip. After the sample step, the platform switched its chamber and injected the initial buffer into the ESSENCE chip at the same speed and amount. Finally, the incubation and rinsing step was set at 5 µL/min for 5 min each. The incubating and rinsing step used a 25 µL sample and initial buffer.

**Figure 1:**
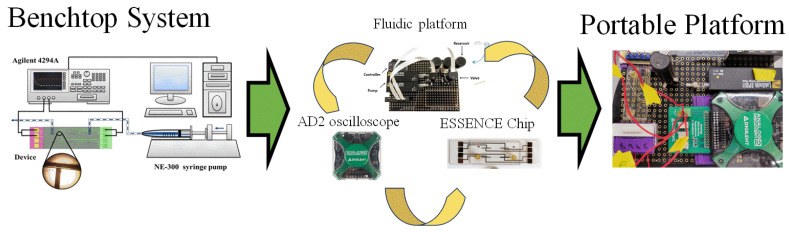
The transition from a benchtop microfluidic system to a portable biosensor system.

A benchtop 4294A impedance analyzer (Keysight Technologies, Santa Rosa, CA, USA) detects target DNA using electrochemical impedance spectroscopy (EIS) with a frequency range from 40 Hz to 100 MHz. The portable Analog Discovery 2 oscilloscope (Digilent, Pullman, WA, USA) was used with an impedance analyzer. Buffers were used to compare the signal sensitivity, ranging from 1 × 10^−4^ M to 1 × 10^−0^ M in the case of KCl, and between 0.1× and 10× PBS. (pH 7.4) for PBS. The concentration of ferricyanide/ferrocyanide pairs used was between 10 mM and 100 mM, while the Tris(bipyridine)ruthenium(II) ([Ru(bpy)_3_]^2+^) used was between 1 mM and 10 mM in concentration. The Nyquist plot from the EIS signal was fitted to a two-electrode equivalent circuit, a traditional Randel circuit, and a modified two-electrode with a Randel circuit by Zview^®^ (Huntington Beach, CA, USA) to obtain the detection signal. The ssDNA concentration in initial tests was 1 µM in different electrolytes. Figure 1 shows the instrument transition in this manuscript, from the benchtop system to an overall portable system. The system contains three major parts: (1) a fluidic platform, (2) an electrochemical analyzer (Analog Discovery 2 oscilloscope), and (3) The ESSENCE chip.

## 3. Results

### 3.1. Impedeimic Equivlent Circuit for Faradic and Non-Faradic Process

**Figure 2:**
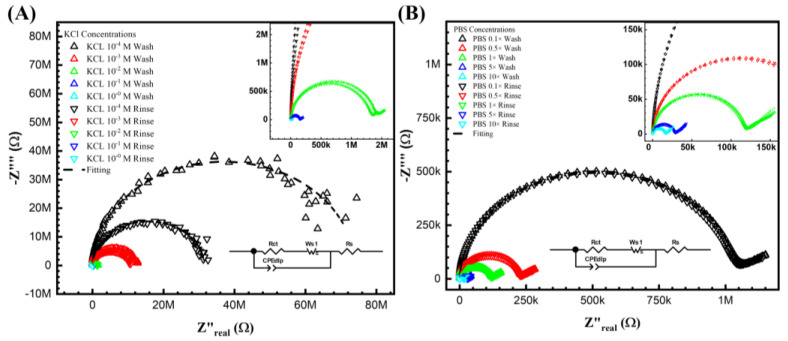
Keysight 4294A response with KCl (**A**,**B**) PBS gradient concentrations from before and after DNA biorecognition bounding. From (**A**) at the lowest concentration $1\times 10^{-4}$ M, the noise can be easily seen from the semicircle, which indicated not only fitting difficulty but also the lowest current limitation of the analyzer. (**B**) The PBS gradient indicated the valued impedance measurements. However, the signal difference between the initial and post-impedance response (wash and rinse) is hardly due to its non-sensitivity system.

The Nyquist plots obtained using Keysight 4294A in KCl and PBS aqueous solutions are shown in Figure 2 after the wash step (no target DNA binding) and the rinse step (post-target DNA binding). The impedimetric data of the non-faradic process show a decreased semicircle radius with increasing electrolyte ionic strength. This inversely proportional relationship is expected. It shows that the ESSENCE chip response to the background solution is accurate. Further, it is also clear that at low concentrations, the difference of the KCl shows a significant difference between the initial and post uM DNA signal; however, there is increased noise at low KCl concentrations. This is due to the current from the chip reaching the Keysight 4294A’s lower bound on measurable current. The noise at the low KCl concentrations makes fitting the data into an equivalent circuit difficult. Hence, obtaining the circuit elements that show the binding of the target to the capture molecule is impossible. Thus, even though the initial/post-impedimetric signal at low concentrations is significant, the lack of fitting makes the data unusable. However, as shown in Figure 2B, the signal from PBS (even at low ionic strength of PBS) has lower charge transfer resistance due to the different molecules used in PBS to balance the pH, namely, NaCl, KCl, Na2HPO4, and KH2PO4. Hence, there were no issues (low noise) encountered in using Keysight 4294A to measure the impediment signal at low PBS concentrations. Both PBS and KCl were fitted with the traditional Randle circuit to determine the charge transfer resistance (Rct values) for further analysis. Though the Randle circuit was described thoroughly elsewhere by us, here, briefly, the circuit elements are Rs, Wo1, CPEdlp, and Rct, which stand for electrolyte and external resistance, Warburg impedance, double-layer capacitance, and surface charge resistance, respectively [37].

**Figure 3:**
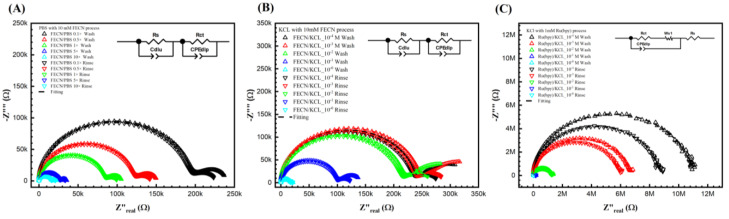
Keysight 4294A response with Faradic process. (**A**) PBS with 10 mM [Fe(CN)_6_]^3−/4−^, (**B**) KCl with [Fe(CN)6]^3−/4−^, and (**C**) KCl with Tris(bipyridine)ruthenium(II) chloride ([Ru(bpy)_3_]^2+^), with gradient concentrations before and after DNA biorecognition bounding. It seems that the redox probe tremendously reduced the resistance in both (**A**,**B**). In (**A**,**B**), the curves were fitted with two parallel equivalent circuits. The circuit at the low frequency indicated the surface change resistance. (**C**) KCl with another redox probe [Ru(bpy)_3_]^2+^ redox. However, it does not show a second semi-curve in the plot. Thus, the fitting was carried out with a regular Randle circuit.

Figure 3B,C show the Nyquist plots for different KCl concentrations with 10 mM ferro/ferricyanide redox probe and 1 mM Tris(2,2′-bipyridine)ruthenium(II) ([Ru(bpy)_3_]^2+^) after the wash step (no target DNA binding) and the rinse step (post-target DNA binding). Unfortunately, it is impossible to obtain a curve of PBS with 1 mM Tris(2,2′-bipyridine)ruthenium(II) ([Ru(bpy)_3_]^2+^)) as the 1 mM Tris(2,2′-bipyridine)ruthenium(II) ([Ru(bpy)_3_]^2+^)) reacts with the PBS. Notably, in Figure 3A,B, a second semicircle was observed at the lower frequency. Figure 3A shows the Nyquist curve for different PBS ionic strengths with a 10 mM ferro/ferricyanide redox probe. Thus, the fitted equivalent circuit for Figure 3A,B consists of two parallel RC circuits in series, as detailed in our previous study and elsewhere [41,42]. Though the double semicircle circuit is described thoroughly elsewhere by us, here, briefly, the circuit elements are Rs, Wo1, CPEdlp, and Rct, which stand for electrolyte and external resistance, Warburg impedance, double-layer capacitance, and surface charge resistance, respectively [37,38,39].

It is important to note that the second semicircle represents the redox process, and the Rct represents the biorecognition signal. However, no second semicircles at lower frequencies are observed in Figure 3C. It can be hypothesized that, unlike the ferro/ferricyanide redox probe, the Tris(2,2′-bipyridine)ruthenium(II) ([Ru(bpy)_3_]^2+^)) does not significantly add to the ionic current (thus high resistance), nor does it then change the charge relaxation times (separation of the RC behavior of the bulk ion from the redox pair leading to the appearance of the two semicircles). Further, in Figure 3C, as the resistance is significantly higher than in Figure 3A,B, the current probably reached 4294A’s current limits, leading to slight noise at lower frequencies. Thus, unlike Figure 3A,B, in (C), the data was fitted to a Randle circuit for better results. Thus, ferro/ferricyanide redox probe was chosen as the probe of interest to optimize further (in terms of ionic and redox strength) to find the correct solvent and redox probe combination for maximum sensitivity.

### 3.2. Buffer Influence of Biorecognition Signal

**Figure 4:**
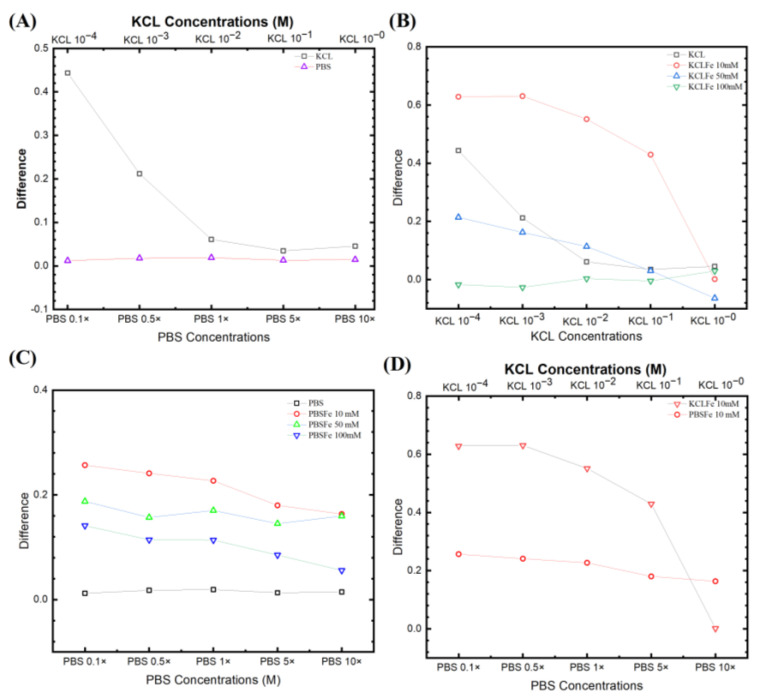
Fitting parameters from different experiential sets. (**A**) Non-faradic comparison for KCl from 10^−4^ to 1 M and PBS from 0.1× to 10×. (**B**) Three different ferro/ferricyanide concentrations paired with different KCl electrolyte concentrations. (**C**) The PBS concentrations with different ferro/ferricyanide redox probe pairs. (**D**) Comparison of signals from the two most responsive pairs for PBS and KCl, with 10 mM [Fe(CN)_6_]^3−/4−^.

Figure 4A compares two non-faradic processes after the wash step (no target DNA binding) and the rinse step (post-target DNA binding). The equivalent circuit was fitted with a traditional Randles circuit (as described above), and the Rct was extracted from the fitting. The KCl provided a higher signal (bigger change in Rct) than in PBS. It is also worth noting that the relevant data from KCl 1 × 10^−4^ M are unreliable due to its intense noise. However, Figure 4A still shows that decreasing the electrolyte concentrations yields a higher difference between initial/post-impedimetric values, that is, delta change in Rct due to the binding of the target DNA. Interestingly for PBS, in the absence of a redox probe, the signal does not change during the initial/post-impedimetric difference. This lowered sensitivity is expected due to the absence of the faradic current generated by the redox probe pairs on the electrode surface, as shown in our earlier studies [37,38,39,43], and elsewhere [44]. Figure 4B shows the KCl with different [Fe(CN)_6_]^3−/4−^ concentrations from 10 mM, 50 mM, to 100 mM. It can be seen that KCl with 10 mM has the highest signal, as also observed by Lacina et al. [32]. Do note that for all KCl concentrations, both faradic and non-faradic, a higher electrolyte concentration results in a lower signal.

Figure 4C shows PBS with different [Fe(CN)_6_]^3−/4−^ concentrations. The electrochemical signal is strongly influenced by the target molecule captured on the electrode surface, where the oxidation and reduction reaction occurred in the redox probe. However, the redox probe, the supporting solution, and the electrolyte contribute to this reactive current. Comparing the EIS signal from both faradic (with redox probe) and non-faradic processes (no redox probe), the non-faradic process has the lowest signal. This is expected due to the absence of the faradic current generated by the redox probe pairs on the electrode surface, as shown in our earlier studies [37,38,39,43] and elsewhere [44]. The EIS signal again also increases with decreasing [Fe(CN)_6_]^3−/4−^ and PBS concentrations. The [Fe(CN)_6_]^3−/4−^ 10 mM shows the highest signal from the biorecognition reaction. Thus, we see that by increasing the electrolyte ion strength, the ESSENCE sensor loses the sensitivity (lower EIS signal) with a concurrent decrease in the overall current (increasing overall Rct). However, if the Rct increases too much, the EIS analyzer cannot capture a corresponding signal due to the noise, as the current is close to the analyzer’s resolution.

**Figure 5:**
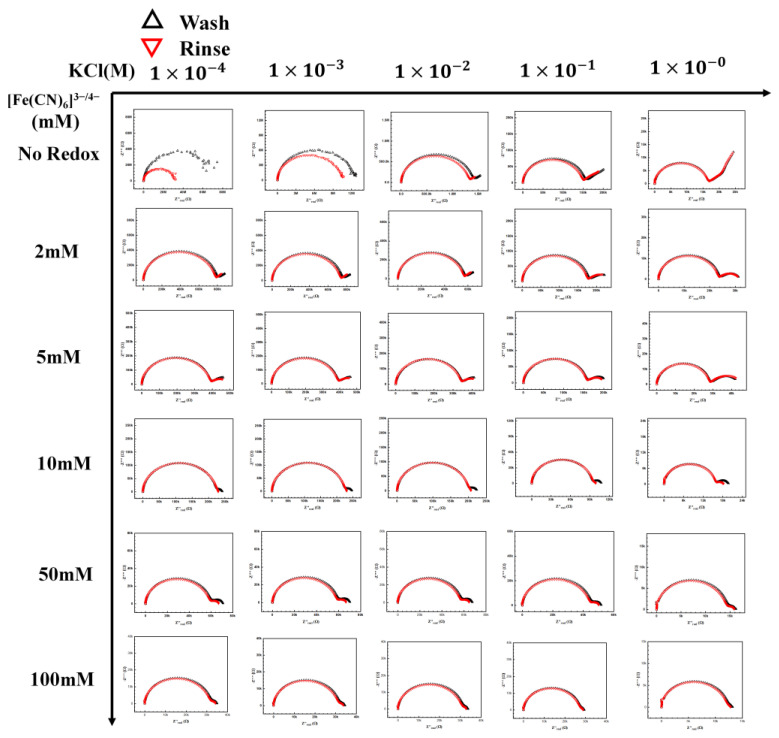
[Fe(CN)_6_]^3−/4−^ redox probe and electrolyte map. A complete map of the redox probe and KCl electrolyte, the redox concentration was tested from no redox to 100 mM with the KCl concentration from 1 × 10^−4^ M to 1 M.

Figure 4D compares the signal from the two most responsive pairs for PBS and KCl, with 10 mM [Fe(CN)6]^3−/4−^. Interestingly for PBS, it has a more gradual slope than the KCl, though it still has the trend that the signal decreases with the increase of the electrolyte. This shows that the redox probe with the lowest concentration has the potential to result in the highest ESSENCE chip response. Thus, multiple ESSENCE chips were run to find the optimized buffer solution for maximum ESSENCE chip response, as shown in Figure 5.

### 3.3. Influence of KCl/[Fe(CN)6]^3−/4−^ Electrolyte/Redox Pair

Figure 5 shows the complete KCl/[Fe(CN)_6_]^3−/4−^ redox map tested in this section. The concentrations of [Fe(CN)_6_]^3−/4−^ redox tested were from 2 mM to 100 mM. Increasing the redox concentration resulted in a second semicircle (due to the redox pair), which is seen at the lower frequencies moving toward the first semicircle, representing the electrolyte. However, a similar movement also occurred for the first semicircle with increasing KCl concentration. It is worth noting that the two semicircles are well separated in the Nyquist plot only at intermittent KCl and [Fe(CN)_6_]^3−/4−^ concentrations. This movement of the redox semicircle can be explained by understanding the effect of the ionic strength of the redox couple and the electrolyte on the capacitance, resistance, and charge transfer relaxation time. The resistance is represented as Equation (1)

(1) $$R=\frac{L}{\sigma A}$$

where σ is the conductivity, L is the length, and A is the material’s area representing the ESSENCE chip’s overall electrode area, including the packing. Furthermore, the capacitance can be calculated as

(2) $$C=\varepsilon \frac{A}{L}$$

where ε represents permittivity, L is the length, and A is the material’s area. The charge relaxation time can be calculated as

(3) $$\tau =\frac{1}{\omega }=RC$$

In this case, we assume that the length and A are the same because they reflect the ESSENCE chip’s structure. Thus, by combining Equations (1) and (2) into (3), we obtain

(4) $$\omega =\frac{1}{RC}=\frac{\sigma }{\varepsilon }$$

**Figure 6:**
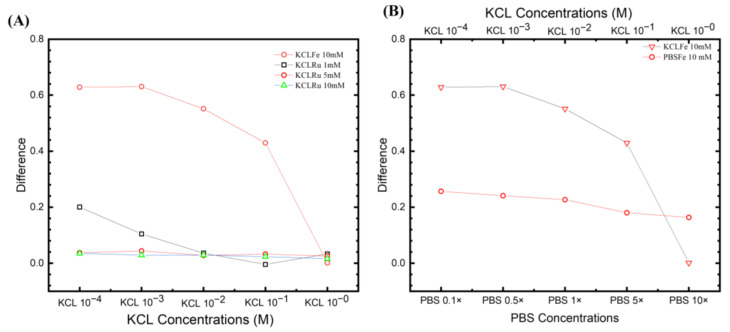
Fitting parameters from different experiential sets. (**A**) The most positive results from the KCl ferro/ferricyanide system with ([Ru(bpy)_3_]^2+^) system. It can be seen that the [Fe(CN)_6_]^3−/4−^ system has a higher ESSENCE chip response. (**B**) The two ferro/ferricyanide systems compared to KCl and PBS, with their highest response. The trend of losing response by increasing the supporting electrolyte response was observed in the figure. However, PBS shows more stability than in KCl.

These equations predict the characteristic charge relaxation frequency, which indicates the approximate frequency range of the semicircle (RC). As the electrolyte’s ionic strength increases, the resistance decreases, as seen in the first row. Hence, an increase in characteristic frequency is observed for the electrolyte, moving the semicircle towards a higher frequency region. However, it is not so straightforward in the presence of the redox ion, as seen in the next few rows. There is an interplay between the redox pair and KCl as the concentrations of both of them change. With the increase in the redox concentration, it is expected that the semicircle will move inward like the electrolyte. However, as can be seen in the middle section in Figure 5, redox pair with concentrations around 1–10 mM and KCl concentration around 1–10 mM, the rate of change in the characterization frequency is different. This makes the redox semicircle distinct from the electrolyte semicircle. Only when the redox pair semic-circle is distinct from the electrolyte semicircle that the maximum change in Rct is observed (highest sensitivity). The distinct semicircle gives us a clear measurement of the change in Rct due to target binding. Thus, a good operational range of KCl concentration is between 1 and 10 mM, with a concentration of 5–10 mM of [Fe(CN)_6_]^3−/4−^ for maximum sensitivity (Figure 6).

### 3.4. Optimized Buffer

**Figure 7:**
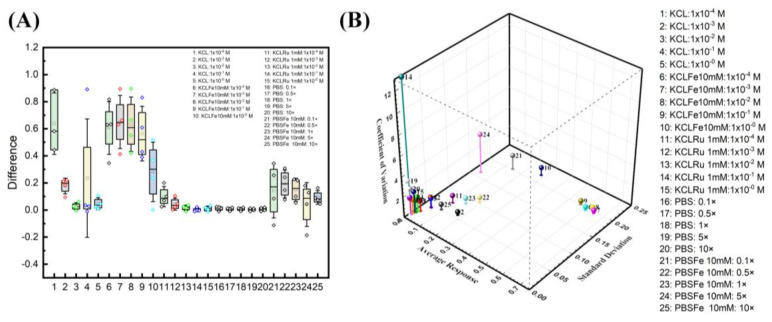
Buffer optimization studies. (**A**) Four ESSENCE chips and five different redox/electrolyte pairs provide the confidential test of the EIS signal response. KCl/[Fe(CN)6]^3−/4−^ pairs have the highest EIS response of all five pairs. The response is around 0.6 to 0.2. The PBS/[Fe(CN)6]^3−/4−^ response is around 0.2 of the normalized signal. The KCl shows a vast difference as observed in the box plot and is unreliable for its lower response fitting with noise. The rest of the KCl/([Ru(bpy)_3_]^2+^) and PBS did not show the EIS response. (**B**) The 3D graph shows the relationship between average response, standard derivation, and coefficient of variation.

Multiple ESSENCE chip was tested to understand the effect of the buffer solutions on the chip-to-chip signal. Four different chips were tested under five different conditions of the redox probe and supporting electrolyte pairs: (1) PBS, KCl; (2) PBS/[Fe(CN)_6_]^3−/4−^, 10 mM; (3) KCl/[Fe(CN)_6_]^3−/4−^, 10 mM; and (4) KCl/([Ru(bpy)_3_]^2+^), 1 mM. The results are shown in Figure 7A. First, KCl and PBS show the same behavior as before for the non-faradic process. The PBS has no EIS signal response before and after the target DNA binding. In the case of KCl, it shows higher signal differences at low concentrations, but it is noisy. On the other hand, the [Fe(CN)_6_]^3−/4−^ redox pairs of PBS and KCl show potential; however, as expected, the KCl/([Ru(bpy)_3_]^2+^) does not show any change in the EIS signal. Comparing KCL and PBS redox pairs, it can be seen that though the KCl/[Fe(CN)_6_]^3−/4−^ has a higher response than the PBS/[Fe(CN)_6_]^3−/4−^, the KCl/[Fe(CN)_6_]^3−/4−^ exhibits a higher noise from the ESSENCE chip in comparison to the PBS/[Fe(CN)_6_]^3−/4−^.

These studies clearly illustrate two significant optimization factors for maximum sensitivity: electrolyte and redox probe concentration. However, choosing a buffer solution is not trivial. For example, choosing a buffer where the difference in Rct between the wash and the rinse steps is the largest is incorrect. We can hypothesize that most of the high resistance values represent the distance between two electrodes, which we plan to optimize later. The enormous resistance value from electrolytes could hinder the minor resistance change from the biorecognition reaction on the electrode surface. Nevertheless, the high Rct change from the buffer is expected to also work for a shorter electrode distance.

Further, the device could be susceptible to any minor disruption in the system. Hence, any buffer with high noise, like KCl, should be avoided. This also mitigates the problem of fitting. Hence, from the optimization results, we find that increasing the electrolyte concentration and lowering the redox probe can separate two semicircles leading to the highest signal with minimal noise. Figure 7B shows the relationship between average response, standard derivation, and coefficient of variation. A combination of higher signal response, lower coefficient of variation, and lower standard deviation is an ideal selection. At first glance, the KCl at lower concentrations (point 2) is a logical choice of all. However, due to the fitting reliability in deficient KCl concentrations, KCl at lower concentrations is not an ideal selection. The closest data sets to the ideal region are divided between the group of KCl and PBS with a 10 mM ferro/ferricyanide redox probe. The KCl region behaves with a higher signal response, but with higher standard deviation and lower coefficients of variation (6–9). On the other hand, the PBS region has slightly higher coefficients of variation, low standard deviation, and average response (22, 23, 25). The KCl region at lower concentrations (6–9) has the issue of fitting reliability. The second semicircle from the EIS response at lower frequencies is not complete. This behavior indicated that the fitted Rct is outward of the acquired data set, hence hindering the equivalent circuit explanation on biorecognition events at the electrode surface.

In this study, we chose the highest conductive pH-stabilized buffer (10× PBS, 25) as it has the lowest standard deviation, a slightly higher coefficient of variation, and an average response. We hypothesized that moving from a costlier impedance analyzer to a cheaper hand-held analyzer would increase the system noise while decreasing the measured current resolution. 22 and 23 are also good choices; however, it has a slightly higher standard deviation, and we value the standard deviation more. Hence, choosing 10× PBS as the buffer of choice would minimize the noise and the standard deviation.

### 3.5. Adapted 4294A Analyzer to a AD2 Portable Device

**Figure 8:**
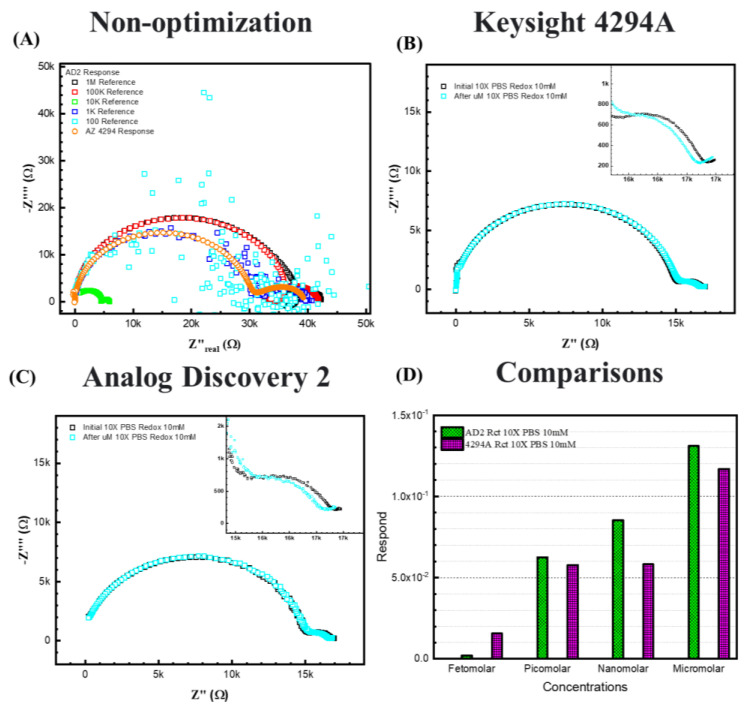
The response between two EIS analyzers. (**A**) shows the case without optimization. (**B**) 10× PBS with 10 mM [Fe(CN)_6_]^3−/4−^redox probes in Keysight 4294A. (**C**) 10× PBS with 10 mM [Fe(CN)_6_]^3−/4−^redox probes in Analog Discovery 2. (**D**) shows the comparisons between 4294A and AD2 results.

Figure 8B,C represent 10× PBS with 10 mM [Fe(CN)_6_]^3−/4−^redox probes, (C) is the response from the AD2 portable EIS analyzer, and (B) is the response from the 4294A benchtop analyzer. For the 10× PBS, due to higher ionic strength in the electrolyte, the semicircle shifted to higher frequencies, resulting in more exposure to the redox semicircle. The Warburg diffusing behavior can be determined at very low frequencies. Thus, to the equivalent circuit of 10× PBS, a Warburg element is added during this fitting to explain the behavior.

Figure 8D shows the fitting results and comparison between the two analyzers for 10× PBS with 10 mM [Fe(CN)_6_]^3−/4−^. The figure shows that for 10× PBS, the two analyzers show similar responses to ssDNA of the same concentrations, except at femtomolar, the 4294A displays a lower response signal. Both analyzers responded to the increasing concentrations, indicating that the portable device is available to make a calibration curve for a cost-effective ESSENCE platform. We expect that decreasing the electrode distance should lead to an enhanced limit of detection even when using the AD2 analyzer.

## 4. Conclusions

This paper demonstrates the interplay between the ionic strength of an electrolyte and the redox buffer and how it can influence the buffer of choice for transitioning from a benchtop EIS analyzer to a portable EIS analyzer. The electrolyte/redox studies show that the increasing and decreasing ionic strength of the supporting electrolyte and the redox probe on the surface response can be significant to the EIS signal of a biosensor. Hence, the correct composition of the surrounding electrolyte and the redox is essential during the POC biosensor development to obtain maximum sensitivity. For the ESSENCE platform, increasing the concentration of the pH-stabilised electrolyte moves the semicircle to the higher frequencies, along with decreasing the [Fe(CN)_6_]^3−/4−^redox probe concentrations that moves the semicircle to the lower frequencies, show the best combination in this study. The changing between two semicircles must also be at the analyzer’s minimum current tolerance, or the signal change is unreliable due to high standard deviation. Choosing a background electrolyte with a minimal standard deviation (like 10× PBS) with a significant redox concentration, like 10 mM [Fe(CN)6]^3−/4−^, allows for transitioning the measurement of the biorecognition signal from the costlier Keysight 4294A to the significantly cheaper Analog Discovery 2. The chosen background buffer solution allows us to detect ssDNA using the Analog Discovery 2, though with a higher detection limit than the Keysight 4294A.

## Data Availability

The data presented in this study are available in article.
